# Prescription Opioids Are Associated With Population Mortality in US Deep South Middle-Age Non-hispanic Whites: An Ecological Time Series Study

**DOI:** 10.3389/fpubh.2019.00252

**Published:** 2019-09-06

**Authors:** Mark A. Brandenburg

**Affiliations:** Department of Medicine, Bristow Medical Center, Bristow, OK, United States

**Keywords:** prescription opioids, population mortality, non-Hispanic whites, US South, US mortality rate

## Abstract

**Objective:** The US Burden of Disease Collaborators reported that between 1990 and 2016, the top 10 states with increasing probability of death between the ages of 20 and 55 years were all in the South. A recent study of annual surveillance data found that increasing all-cause mortality rates were occurring in middle-age non-Hispanic whites. The vast proportion of all-cause mortality consists of medical causes, not external causes (i.e., overdose, mental illness, suicide, homicide, or motor vehicle crashes). It has been hypothesized by researchers that the ongoing opioid epidemic has an etiologic role in the trend of increasing medical death, but ecological studies looking for an association have not been published. The objective of this study was to test the hypothesis that hydrocodone and oxycodone sales are temporally associated and correlated with annual NHW45-54 medical-cause mortality rates in the Deep South region comprised of Alabama, Arkansas, Louisiana, Mississippi, Oklahoma, and South Carolina.

**Methods:** Mortality and opioid sales data were obtained from the Centers for Disease Control and Prevention Wonder Detailed Mortality and University of Wisconsin State Health Access Data Assistance Center databases, respectively. Annual, state and regional NHW45-54 medical-cause mortality and opioid sales data were analyzed using Spearman rank correlation (rs) testing, after first and second differencing, in order to achieve stationarity and control for trend similarities.

**Results:** Sales of prescription opioids follow very similar temporal patterns across these six states, with simultaneous increases in 2007 and 2013. With few exceptions, annual opioids sales trends were correlated state-to-state. Two prominent spikes are evident in the aggregated opioid sales trends of the six states, with both sales spikes preceding same-directional fluctuations in medical-cause mortality by ~1 year. After a 1 year adjustment of second-differenced data, population hydrocodone exposure was correlated with female NHW45-54 population medical-cause mortality [rs_(13)_ = 0.540; *P* = 0.038]; and oxycodone exposure correlated with male NHW45-54 population medical-cause mortality [rs_(13)_ = 0.607; *P* = 0.016].

**Conclusions:** State sales of prescription hydrocodone and oxycodone in the six states studied follow non-random, systematic trajectories. A strong correlation and temporal association exists between prescription opioid sales and medical-cause mortality in this Deep South NHW45-54 population.

## Introduction

Life expectancy worldwide has generally been increasing for more than 100 years (1). However, a recent US ecological time-trend study of 1999–2013 annual surveillance data found increasing all-cause mortality rates in middle-age non-Hispanic whites, while in other racial groups all-cause mortality rates are decreasing (2). Associations with widening income inequality and economic insecurity, prescription opioids and abuse, suicide, alcoholism, and cirrhosis have all been considered—yet, the etiology of rising all-cause mortality remains elusive.

Between 1990 and 2016, the top 10 states with increasing probability of death between the ages of 20 and 55 years were all in the South. These states were: Alabama, Arkansas, Kentucky, Louisiana, Mississippi, New Mexico, Oklahoma, South Carolina, Tennessee, and West Virginia. The greatest proportion of all-cause mortality in these states is comprised of medical disease, not external causes (i.e., overdose, mental illness, suicide, homicide, or motor vehicle crashes) (3). Given that opioid prescribing has also been on the rise (4), and is greatest in the South (5), and that the 45–54-year-old age group also suffers the highest drug- and alcohol-related mortality rates (6), prescription opioid exposure is a candidate risk factor for increasing medical-cause mortality in this region.

Most studies measuring associations between prescription opioids and mortality are small, and focus on individual exposure and overdose outcomes. The diversion of prescribed opioids into the broader, non-patient population is commonplace (7)—thus, prescription opioid exposure is a population phenomenon (8). Ecological studies are necessary to test the hypothesis of an association between prescription opioids and US population mortality trends. Studies, however, looking for an association between population exposure to prescription opioids and medical-cause mortality rates are lacking.

The aim of this study was to determine whether population exposure to prescription opioids in the South is a risk factor for increasing NHW45-54 medical-cause mortality (i.e., death from cardiovascular, pulmonary, gastrointestinal, infectious, and most other medical diseases). The objective was to test the hypothesis that aggregated hydrocodone and oxycodone sales are temporally associated and correlated with annual NHW45-54 medical-cause mortality rates in the Deep South region comprised of Alabama, Arkansas, Louisiana, Mississippi, Oklahoma, and South Carolina.

## Methods

### Study Population

In a previous study, mortality datasets from Kentucky, Tennessee, New Mexico, and West Virginia failed to meet criteria for stationarity using Augmented Dickey-Fuller testing (i.e., *P*-value >0.05) after first-differencing; therefore, data from these states were dropped from the current study (9). The remaining six Deep South states of Alabama, Arkansas, Louisiana, Mississippi, Oklahoma, and South Carolina were selected for study.

### Databases

Annual medical-cause mortality data were obtained from the publicly accessible Centers for Disease Control and Prevention (CDC) Wonder Detailed Mortality database (10). The International Classification of Diseases (ICD-10) codes published by the World Health Organization were relied upon for cause-specific mortality classifications. Medical-cause mortality was defined in Table 6 of the CDC Wonder Detailed Mortality database as **all categorical causes of death except F01-F99 (Mental and behavioral disorders) and V01-Y89 (External causes of morbidity and mortality)**. The following cause-of-death categories were included in the dataset for analyses:

A00-B99 (Certain infectious and parasitic diseases)
C00-D48 (Neoplasms)
D50-D89 (Diseases of the blood and blood-forming organs and certain disorders involving the immune mechanism)
E00-E88 (Endocrine, nutritional, and metabolic diseases)
G00-G98 (Diseases of the nervous system)
H00-H57 (Diseases of the eye and adnexa)
H60-H93 (Diseases of the ear and mastoid process)
I00-I99 (Diseases of the circulatory system)
J00-J98 (Diseases of the respiratory system)
K00-K92 (Diseases of the digestive system)
L00-L98 (Diseases of the skin and subcutaneous tissue)
M00-M99 (Diseases of the musculoskeletal system and connective tissue)
N00-N98 (Diseases of the genitourinary system)
O00-099 (Pregnancy, childbirth, and puerperium)
P00-P96 (Certain conditions originating in the perinatal period)
Q00-Q99 (Congenital malformations, deformations, and chromosomal abnormalities)
R00-R99 (Symptoms, signs, and abnormal clinical and laboratory findings, not elsewhere classified)
U00-U00 (Codes for special purposes)

By de-selecting the two broad categories of external-cause mortality from the dataset (i.e., mental/behavioral disorders, and traumatic causes), overdoses, depression, suicide, homicide, and other unintentional traumatic deaths were excluded from the data analyses. The categories of 45–54 years of age and non-Hispanic white mortality were selected in the CDC database. Mortality rates are presented as deaths per 100,000. All data were anonymized by the database owners prior to this project. Mortality data were not used in the final models if suppressed by CDC, due to sparseness.

Annual hydrocodone and oxycodone sales data in kilograms per 100,000 persons were collected from the publicly accessible University of Wisconsin State Health Access Data Assistance Center (SHADAC) database; the data of which originated at the US Drug Enforcement Agency's Automated Reports and Consolidated Ordering System (ARCOS) Retail Drug Summary Reports (11).

### Time-Series Data Testing and Analyses

All data collected were trending upward from 2000 to 2017 (see Results section for statistical results). Augmented Dickey-Fuller (ADF) tests were performed with trend in the regression and a lag of one. Aggregated state medical-cause mortality datasets met criteria for stationarity using ADF testing (i.e., MacKinnon *P*-value < 0.05); after first-differencing. All individual state hydrocodone sales datasets, after first-differencing, failed to meet criteria for stationarity using ADF testing (i.e., MacKinnon *P*-value >0.05); but after second-differencing, criteria for stationarity were met by all except Arkansas and Oklahoma. The six-state aggregated hydrocodone sales dataset also required second-differencing to achieve stationarity. State oxycodone sales datasets, after first-differencing, failed to meet criteria for stationarity using ADF testing (i.e., MacKinnon *P*-value >0.05); therefore, second-differencing of the datasets was undertaken; with all but Mississippi and Oklahoma datasets meeting criteria for stationarity. The six-state aggregated oxycodone sales dataset also required second-differencing to achieve stationarity.

Stata 15.1 software was used to generate graphs and perform statistical calculations. Annual, state and regional NHW45-54 medical-cause mortality and opioid sales datasets were analyzed using Spearman rank correlation (rs) testing, after second differencing, in order to achieve stationarity and control for increasing trend similarities. Mortality time series graphs were generated to demonstrate trend and period effect comparisons between populations. Graphs that compare mortality and opioid sales time series period effects utilize a dual-Y-axis format automatically produced by Stata 15.1, in order to compare period effects of variables with different measurement units.

## Results

### State Medical-Cause Mortality Rates

**Figure 1:**
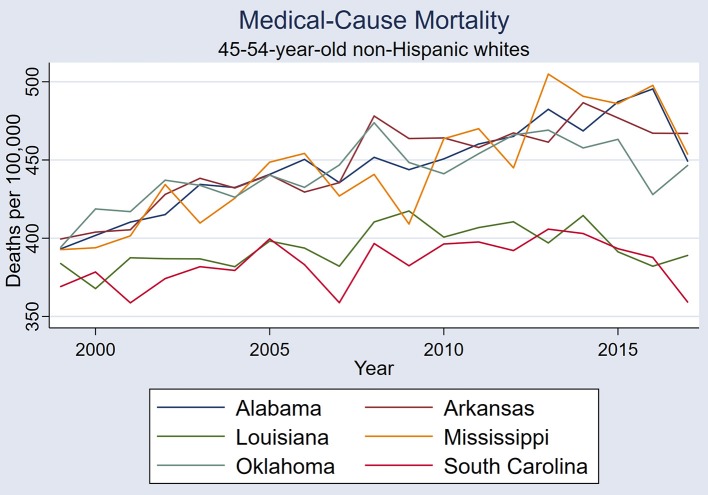
Medical-cause mortality trends in the six states: Alabama, Arkansas, Louisiana, Mississippi, Oklahoma, and South Carolina.

**Figure 2:**
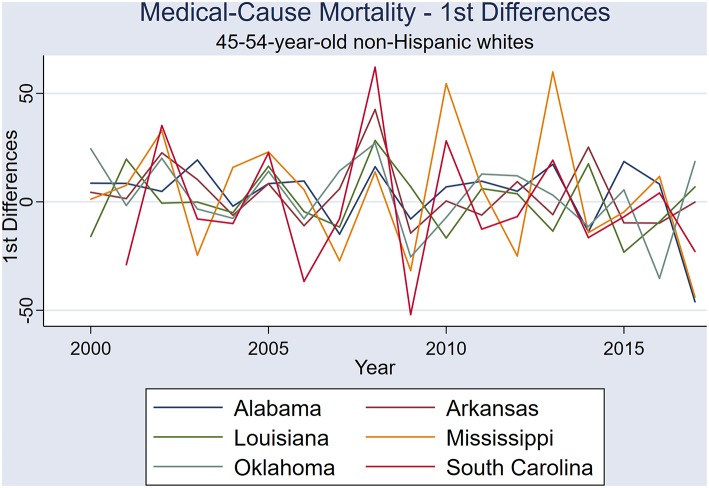
First differences of annual medical-cause mortality rates in the six states: Alabama, Arkansas, Louisiana, Mississippi, Oklahoma, and South Carolina.

**Figure 3:**
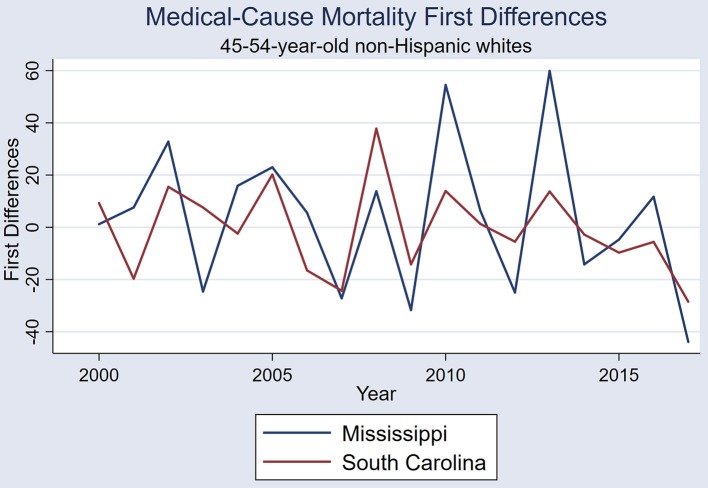
First differences of annual medical-cause mortality rates in Mississippi and South Carolina (Spearman rank coefficient 0.701, *P* = 0.001).

The six states showed similar medical-cause mortality rate period effects, with similar period effects in 2005, 2008, and 2013-4 (Figure 1). A visual comparison of first differences exemplifies the similarities in mortality rate period effects between these six states (Figure 2). One example of states with correlated medical-cause mortality time series is the Mississippi & South Carolina pair [rs_(16)_ = 0.701, *P* = 0.001], using first-differences (Figure 3).

**Figure 4:**
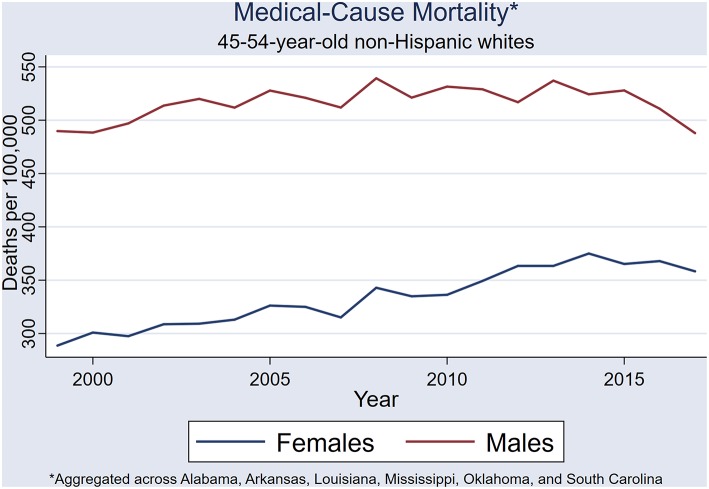
State-aggregated (Alabama, Arkansas, Louisiana, Mississippi, Oklahoma, and South Carolina) medical-cause mortality trend.

The six-state-aggregated medical-cause mortality time series increased during the first 10 years for both females and males (i.e., 1999–2008); however, the gender-specific trends diverged after 2008 (Figure 4). Male medical mortality was stable from 2009 until 2015 when a precipitous decline occurred. Across the whole study period (i.e., 1999–2017), male medical mortality did not exhibit a trend [rs_(16)_ = 0.3298, *P* = 0.168], whereas female mortality rate continued to climb.

Female medical mortality continued to rise throughout the study period (i.e., 1999 through 2017), and an overall upward trend was demonstrated [rs_(16)_ = 0.954, *P* < 0.001] (Figure 4). In the female NHW45-54 population, a 19% increase in medical-cause mortality occurred from 1999 to 2008, with four prominent rate spikes observed: 2002, 2005, 2008, 2014. In a single year, from 2007 to 2008, medical-cause mortality increased by ~9%. A steady increase of 12% in medical-cause mortality rates occurred from 2009 to 2014, followed by a decline of 4.5% by 2017. Very similar period effects are seen in both male and female medical mortality time series.

### State Prescription Opioid Sales

**Figure 5:**
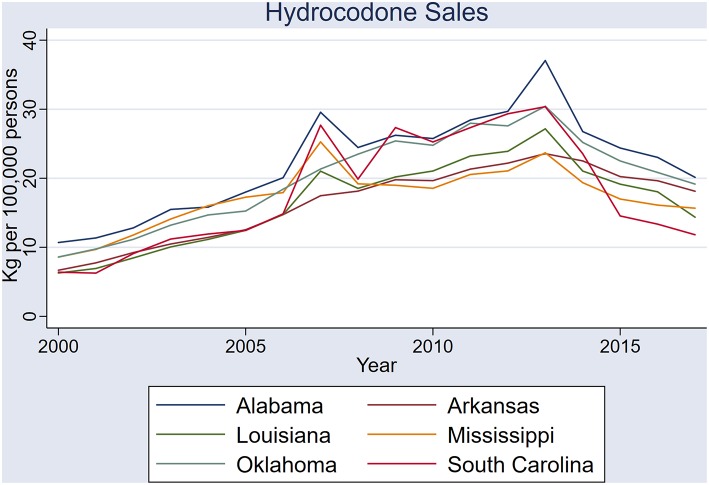
Sales of hydrocodone (kilograms per 100,000 persons) in the six states: Alabama, Arkansas, Louisiana, Mississippi, Oklahoma, and South Carolina.

State sales of prescription hydrocodone followed very similar, upward trends in all six states (Table 1), with simultaneous sales spikes in 2007 and 2013 (Figure 5). Correlations were strong in all pairs of states, except Oklahoma and South Carolina (Table 1).

**Table 1:** Annual hydrocodone sales time series Spearman rank correlations between the six states, using second differences (2000–2017).

	**Year (test for trend)**	**AL**	AR^*^	**LA**	**MS**	OK^*^	**SC**
**AL**	0.959 *P* < 0.001	1.000					
AR^*^	0.965 *P* < 0.001	0.577 *P* = 0.019	1.000				
**LA**	0.994 *P* < 0.001	0.915 *P* < 0.001	0.659 *P* = 0.006	1.000			
**MS**	0.880 *P* < 0.001	0.806 *P* < 0.001	0.621 *P* = 0.010	0.818 *P* = 0.001	1.000		
OK^*^	0.963 *P* < 0.001	0.571 *P* = 0.021	0.659 *P* = 0.006	0.662 *P* = 0.005	0.612 *P* = 0.012	1.000	
**SC**	0.959 *P* < 0.001	0.550 *P* = 0.027	0.850 *P* < 0.001	0.556 *P* = 0.025	0.606 *P* = 0.013	0.347 *P* = 0.188	1.000

*Data are from the CDC Wonder Detailed Mortality databases of the six study states: Alabama (AL), Arkansas (AR), Louisiana (LA), Mississippi (MS), Oklahoma (OK), and South Carolina (SC)*.

* *Arkansas and Oklahoma datasets did not meet criteria for stationarity using Augmented Dickey-Fuller testing (i.e., MacKinnon P-value > 0.05), after second differencing. The mortality data was not differenced in the Test for Trend*.

**Figure 6:**
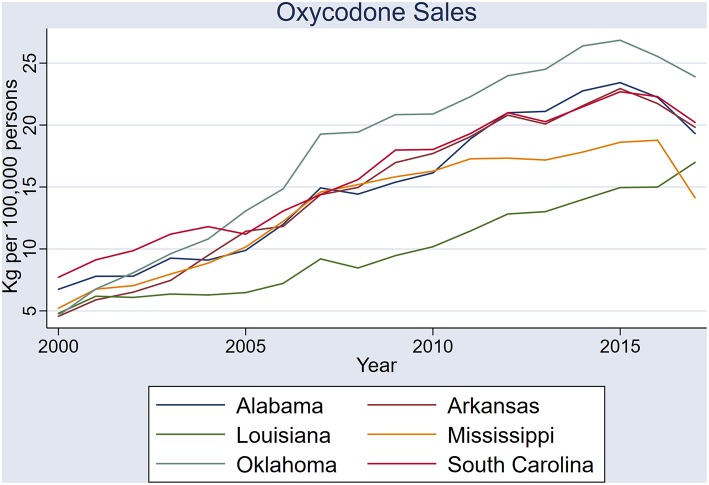
Sales of oxycodone (kilograms per 100,000 persons) in the six states: Alabama, Arkansas, Louisiana, Mississippi, Oklahoma, and South Carolina.

Sales of prescription oxycodone also had similar period effects across the six states, but with greater variability (Figure 6). Common to all states was a spike in oxycodone and hydrocodone sales in 2007, whereas a similar 2013 oxycodone spike was not evident. Among the three states in which oxycodone time series datasets met criteria for stationarity, all pairs showed correlation, except Louisiana and South Carolina (Table 2).

**Table 2:** Annual oxycodone sales time series Spearman rank correlations between the six states, using second differences (2000–2017).

	**Year (test for trend)**	AL^*^	**AR**	**LA**	MS^*^	OK^*^	**SC**
AL^*^	0.959 *P* < 0.001	1.000					
**AR**	0.965 *P* < 0.001	0.668 *P* = 0.005	1.000				
**LA**	0.994 *P* < 0.001	0.662 *P* = 0.005	0.615 *P* = 0.011	1.000			
MS^*^	0.880 *P* < 0.001	0.818 *P* < 0.001	0.488 *P* = 0.055	0.465 0.070	1.000		
OK^*^	0.963 *P* < 0.001	0.750 *P* < 0.001	0.862 *P* < 0.001	0.756 *P* < 0.001	0.538 *P* = 0.032	1.000	
**SC**	0.959 *P* < 0.001	0.709 *P* = 0.002	0.494 *P* = 0.052	0.432 *P* = 0.094	0.627 *P* = 0.009	0.447 *P* = 0.083	1.000

*Data are from the CDC Wonder Detailed Mortality databases of the six study states: Alabama (AL), Arkansas (AR), Louisiana (LA), Mississippi (MS), Oklahoma (OK), and South Carolina (SC)*.

* *Alabama, Mississippi and Oklahoma datasets did not meet criteria for stationarity using Augmented Dickey-Fuller testing (i.e., MacKinnon P-value > 0.05), after second differencing. The mortality data was not differenced in the Test for Trend*.

**Figure 7:**
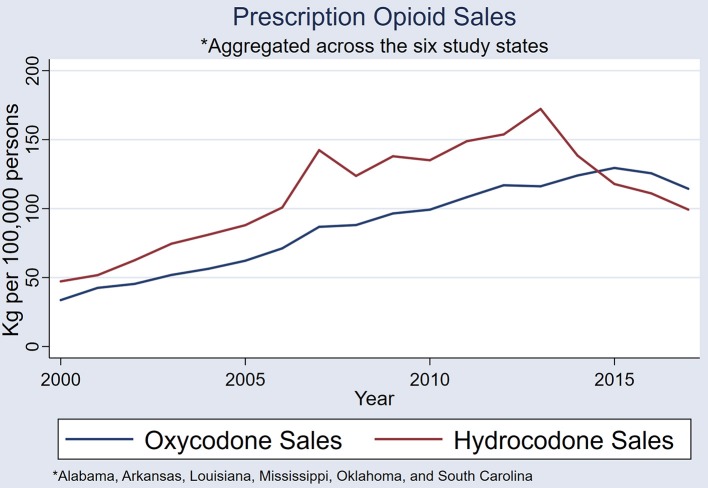
A comparison of state-aggregated (Alabama, Arkansas, Louisiana, Mississippi, Oklahoma, and South Carolina) hydrocodone and oxycodone sales (kilograms per 100,000 persons).

The six-state-aggregated, annual sales of prescription hydrocodone and oxycodone exhibited upward trends: [rs_(15)_ = 0.655, *P* = 0.003] and [rs_(15)_ = 0.965, *P* < 0.001], respectively. Hydrocodone sales spiked in 2007 and 2013, and oxycodone sales also spiked in 2007 (Figure 7).

### Association Between Prescription Opioid Sales and Population Mortality

**Figure 8:**
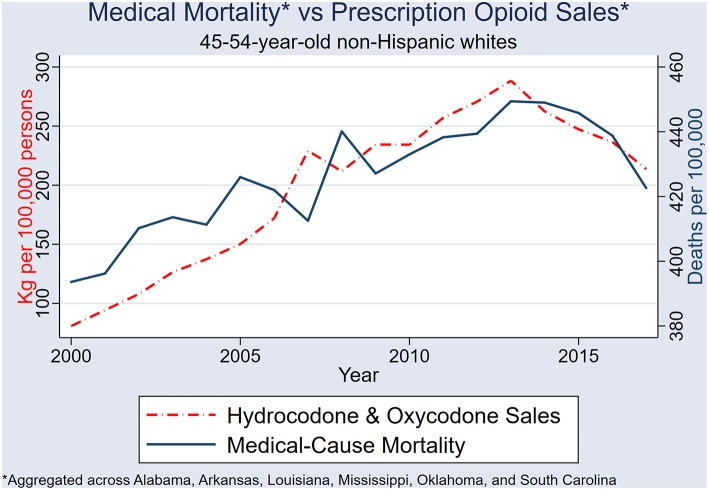
Sales of combined hydrocodone-oxycodone (kilograms per 100,000 persons) and NHW45-54 medical-cause mortality in the six states: Alabama, Arkansas, Louisiana, Mississippi, Oklahoma, and South Carolina.

When the six state datasets are aggregated, male and female mortality rates combined, and annual sales of hydrocodone and oxycodone combined, the 2007 and 2013 spikes in opioid sales each very closely precede same-directional fluctuations in medical-cause mortality by ~1 year (Figure 8).

**Figure 9:**
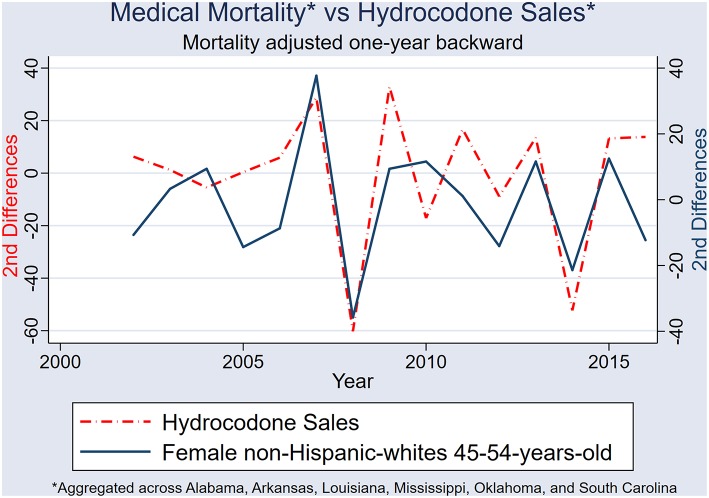
Second differences of state-aggregated, annual hydrocodone sales and female NHW45-54 medical-cause mortality, using a backward adjustment of time series by 1 year.

**Figure 10:**
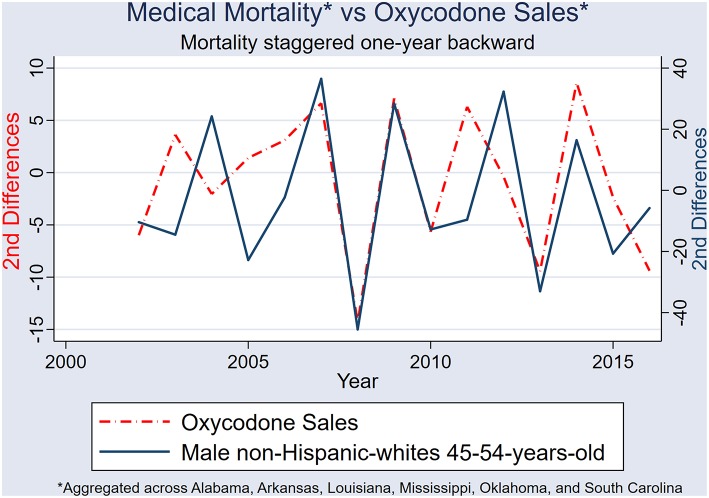
Second differences of state-aggregated, annual oxycodone sales, and male NHW45-54 medical-cause mortality, using a backward adjustment of time series by 1 year.

After 2nd differencing both datasets (to achieve time series stationarity, and control for trend), and adjusting the medical-mortality datasets 1 year back (to control for temporality), population hydrocodone exposure was correlated with female NHW45-54 medical-cause mortality [rs_(13)_ = 0.540; *P* = 0.038] (Figure 9); and oxycodone exposure correlated with male NHW45-54 medical-cause mortality [rs_(13)_ = 0.607; *P* = 0.016] (Figure 10). Correlations were not seen between hydrocodone sales and male mortality [rs_(13)_ = 0.257; *P* = 0.355], or oxycodone sales and female mortality [rs_(13)_ = 0.075; *P* = 0.790].

## Discussion

This is the first study that attempts to demonstrate a correlation between population exposure to prescription opioids and population medical-cause mortality. The time series correlations, and temporal association of period effects between opioid sales and NHW45-54 medical-cause mortality rates indicate that prescription opioids are a risk factor for rising medical-cause mortality in this population. This study controls for trend in the exposure and outcome variables, and it is therefore unlikely that the correlations are coincidental or spurious. Individual-level studies will be necessary to determine whether the rising medical mortality in the South exists due to actual medical disease, and which diseases; or represents misclassification of death (i.e., substantial numbers of patients dying of opioid overdose recorded as having died of medical conditions).

### The Opioid Epidemic

The United States is in the midst of an historic opioid epidemic that began in the 1990s (12–14), driven by inappropriate prescribing practices of physicians (15), during a time in which opioid prescribing (16), and healthcare spending for pain-related conditions increased substantially (17). Prescription opioid use is known to be associated with overdose and other external-causes of death (e.g., depression, suicide, homicide, and motor vehicle crashes). In one cohort study, 9,940 patients who received 100 or more morphine milligram equivalents per day over a 90-day period for chronic noncancer pain, were found to have a 8.9-fold increase in overdose risk (18). Higher dosages of opioids (19), and long-acting opioids (20) have been found to be associated with various causes of drug-related mortality. At the state-level in North Carolina, a temporal trend in the sales of prescription opioids correlated with temporal trends in patient emergency department visits for opioid overdoses (21).

### Opioids and Medical Illness

Various medical co-morbidities at the individual-level are known to be associated with opioid abuse and addiction: infections (e.g., endocarditis, osteomyelitis, septic arthritis, or epidural abscess) (22), immune compromise (23), and sleep-disordered breathing (24). Preoperative prescription opioid abuse and dependence is associated with in-hospital, all-cause mortality after orthopedic surgery (25).

Opioid use disorder is defined as a problematic pattern of opioid use leading to clinically significant impairment or distress (26, 27), and includes a broad range of symptoms and behaviors that can result in death secondary to exacerbations of medical conditions, masking of symptoms, or non-compliance. Opioids taken in escalating doses can lead to respiratory depression and death in patients with lung disease; opioids can mask chest pain in patients with acute coronary syndrome (i.e., heart attack); opioids can lead to trauma that results in medical complications such as infection; and the craving of opioids might distract patients enough that they become noncompliant with other prescribed medications or treatments. These are just a few examples, with many other mechanisms of medical mortality in patients taking prescription opioids likely to exist.

### Population Opioid Exposure and Medical-Cause Mortality

Sales of hydrocodone, and to a lesser extent oxycodone, in the six study states have simultaneous spikes, and appear to have been systematic across the region. All but one state pair in the six states exhibited strong correlations in hydrocodone sales—clear indication that these fluctuations are non-random. Determining the cause of these non-random sales patterns will be important in the planning of future health care policies in the US. An examination of government policies, healthcare management plans, and prescribing practices in these Deep South states will give more information on the systematic nature of sales across the states.

Major fluctuations in opioid sales are closely followed by same-directional trajectory changes in medical-cause mortality, by ~1 year. The first two spikes in NHW45-54 medical-cause mortality occurred during a rapid escalation in Medicaid opioid prescribing, which could have modified the effect between overall opioid sales and mortality prior to 2006. It has been demonstrated that prescription opioid use is more prevalent in patients with public insurance (28). Researchers have hypothesized that exposure to Medicaid-funded healthcare is a risk factor for increasing mortality in the 45–54 population due to the over-prescribing of opioids (29, 30).

### Population Opioid Exposure and Female Mortality

This study demonstrates that hydrocodone sales are associated with female medical-cause mortality, and oxycodone sales are associated with male medical-cause mortality. After 2008, female mortality in the study population continued to increase while male mortality remained stable and then decreased. In 2013, another prominent spike in hydrocodone sales is observed, but not oxycodone sales, when female mortality rates continued to rise. It has been shown that prescription opioid use is more common in females (31, 32), and this study demonstrates a strong correlation between female mortality rates and population exposure to hydrocodone.

### Population Mortality Risk Factors

Several other known mortality risk factors have been hypothesized to be etiologic of increasing mortality in the US. Smoking, obesity, and poverty all have known associations with population mortality, but these risk factors have not been linked to the current increasing mortality or the time series period effects. Poverty in the deep South has been rising, but not with great variability. One must also consider the possibility that prescription opioids are an upstream determinant of unemployment and poverty.

Smoking is well-known to be associated with poverty and mortality, and has been hypothesized to be a possible cause of rising mortality. But, smoking rates have not increased in the past 20 years, and dramatic year-to-year fluctuations certainly not reported. The delayed uptake (i.e., later than males) of smoking decades ago by women could be one reason medical-cause mortality is increasing in women, but this would not hold true for rising male mortality from 1999 to 2008. Southern states have seen an increased prevalence of obesity in the last 20 years, and increasing morbidity and mortality of obesity-related diseases might follow. However, once again, the impact of obesity on population mortality will not likely lead to mortality time series period effects with such non-random variability, as seen between categories of mortality, states, and genders. The dissemination of massive quantities of prescription opioids into this region is the most plausible risk factor, as it is without the aforementioned arguments.

### Limitations

No doubt, the etiology of increasing middle-age mortality in the US is multifactorial. Epidemiologists must consider the analytical challenges of confounding and effect modification. This ecological study is no exception, and certainly cannot draw conclusions about causality. Moreover, with the well-known public health issues of smoking and obesity in the Deep South, the population burden of prescription opioids is likely to be one of a few risk factors associated with increasing mortality. However, it is also possible that smoking and obesity are confounders or even upstream determinants of an association between opioids and population mortality. Indeed, smoking, obesity, and poverty are all upstream determinants of health care exposure; and prescription opioids are disseminated into the population through the healthcare system. Large, detailed, case-control and cohort studies in the future will be necessary to better understand the interaction and confounding between these known risk factors.

### Mortality Misclassification

Measurement error in this study could be responsible for the association between population opioid exposure and medical-cause mortality. Cause of death determinations are often made by primary care physicians, and it is plausible that information bias (physician reporting error) might lead opioid-prescribing physicians to report medical-cause of death determinations when in fact overdoses or other opioid-associated illnesses are responsible for patient death. This direction of misclassification is a particular concern in the current climate of physician arrests for over-prescribing opioids (33, 34). If, indeed, misclassification is present to such an extent that major upward shifts in medical-cause mortality rates are seen, then current estimates of opioid overdose death rates are vastly underestimated.

### Future Research

Specifically, individual data on opioid prescribing, gender, medical examiner reports, and more specific geographical locations of the deceased would allow for case-control and cohort studies that could corroborate ecological study results and better characterize increasing all-cause mortality. More precise measurements could also be made using individual data in cohort studies of opioid prescriptions and mortality. Prescribing data from the state Medicaid programs, Medicare, Drug Enforcement Agency, and Medical Examiner's Offices, would allow closer examination of physicians who are overprescribing opioids to see if they are associated with geographic hotspots of mortality.

## Conclusions

Opioid sales across the six states of Alabama, Arkansas, Louisiana, Mississippi, Oklahoma, and South Carolina have very similar period effects, with strong correlations between most state pairs. Sales of prescription hydrocodone and oxycodone across these six states were systematic, and not random. A strong temporal association exists between population exposure to prescription hydrocodone and oxycodone and NHW45-54 medical-cause mortality. Hydrocodone exposure is associated with female NHW45-54 medical-cause mortality, and oxycodone exposure with male NHW45-54 medical-cause mortality.

## Data Availability

The datasets generated for this study are available online: https://wonder.cdc.gov/controller/datarequest/D76 (10), http://statehealthcompare.shadac.org/ (11).

## Author Contributions

MB is the sole researcher and author of this paper, and conceived of the project design, conducted the background work, collected the data, performed all statistical analyses, and wrote the manuscript. This work is an extension of his Masters in Epidemiology research project while at the London School of Hygiene and Tropical Medicine, but is not the original project submitted.

### Conflict of Interest Statement

The author declares that the research was conducted in the absence of any commercial or financial relationships that could be construed as a potential conflict of interest.
